# 

**Published:** 1912-06

**Authors:** 


*The following donations have been received since the publication of the List in March.*

*May 31st*, 1912.

| | |
|---|---|
| Dr. D. A. Alexander (1) .. .. .. .. | 2 volumes. |
| J. Paul Bush, C.M.G. (2) .. .. .. .. | 2 ,, |
| L. M. Griffiths (3) .. .. .. .. | 14 ,, |
| The Middlesex Hospital (4) .. .. .. | 2 ,, |
| The Surgeon-General, United States Army (5) | 1 volume. |

A framed picture has been presented by Mr. L. M. Griffiths.

### EIGHTY-FOURTH LIST OF BOOKS.

The titles of books mentioned in previous lists are not repeated.

The figures in brackets refer to the figures after the names of the donors, and show by whom the volumes were presented. The books to which no such figures are attached have either been bought from the Library Fund or received through the *Journal*.

| | | |
|---|---|---|
| **Ballance, C. A.** .. | *Cerebral Decompression in Ordinary Practice* .. | 1912 |
| **Beaven, A. B.** .. | *Bristol Lists* .. .. .. .. .. .. .. .. (3) | 1899 |
| **Begg, C.** .. .. | *Sprue: its Diagnosis and Treatment* .. .. | 1912 |
| **Berry and T. P. Legg, J.** | *Harelip and Cleft Palate* .. .. .. .. .. | 1912 |
| **Boyle, H. E. G.** .. | *Practical Anæsthetics* .. .. .. .. .. 2nd Ed. | 1911 |
| **Bryce, J.** .. .. .. | *The Plague of London* .. .. .. .. .. .. (3) | 1860 |
| **Burggraeve, A.** .. | *Livre d'Or de la Médecine Dosimetrique* .. (3) | 1886 |
| **Burghard, Sir W. W. Cheyne and F. F.** | *A Manual of Surgical Treatment.* New Edition. (Revised by T. P. Legg and A. Edmunds.) .. .. Vol. II. | 1912 |

**Burnet, E.** .. .. *Microbes and Toxins* (Trans. by C. Broquet and W. M. Scott) .. .. .. .. .. .. 1912
**Bury, J. S.** .. .. *Diseases of the Nervous System* .. .. .. .. .. 1912
**Cameron, Sir H. C.** *On the Evolution of Wound-Treatment* .. (2) 1907
**Candy, H. C. H.** .. *A Manual of Physics* .. .. .. .. .. .. 1911
*Catalogue (Index-) of the Library of the Surgeon-General's Office, United States Army.* 2nd Ser., Vol. XVI. (5) 1911
**Cayley, W.** .. .. *Pathology and Treatment of Typhoid Fever* (3) 1880
**Cheyne and F. F. Burghard, Sir W. W.** *A Manual of Surgical Treatment.* New Edition (Revised by T. P. Legg and A. Edmunds) .. .. .. Vol. II. 1912
**Clarke, J. J.** .. .. *Protozoa and Disease.* Pt. III. .. .. .. 1912
**Crowe, H. W.** .. *Consumption : Home Treatment and Rules for Living* .. .. .. .. .. .. .. .. 3rd Ed. 1912
**Elizalde, P. I.** .. *Pancreatitis acuda hemorrágica* .. .. .. 1911
*Examination Papers (Edinburgh), Dental* .. .. .. .. .. .. .. [N.D.]
*Examination Papers (Edinburgh), Fellowship* .. .. .. .. .. .. 1912
**Fernie, W. T.** .. *Health to Date* .. .. .. .. .. .. .. .. .. 1911
**Forbes, J. G.** .. .. *The Pollution of Swimming-Baths* .. .. .. 1912
**French, H.** (Ed.) .. *An Index of Differential Diagnosis* .. .. .. 1912
**Gebele, H.** .. .. *Die Chirurgischen Untersuchungsmethoden* .. 1912
**Harris, A.** .. .. *Etiology, Diagnosis and Prophylaxis of Phthisis* 1912
**Harrison, C. E. Pollock and L. W.** *Gonorrhœal Infections* .. .. 1912
*Homes of the Bristol Poor, The* .. .. .. .. .. .. .. .. .. .. (3) 1884
**Hutchison and H. Rainey, R.** *Clinical Methods* .. .. 5th Ed. 1912
**Jamieson, W. A.** .. *The Care of the Skin in Health* .. .. .. 1912
**Johnson, S.** .. .. *A Dictionary of the English Language.* 2 vols. (1) 4th Ed. 1775
**Keith, Sir F. Treves and A.** *Surgical Applied Anatomy* .. 6th Ed. 1911
**Krefting, R.** .. .. *Der Wasserman'ske Reaktion* .. .. .. .. .. 1911
**Legg, J. Berry and T. P.** *Harelip and Cleft Palate* .. .. .. .. .. 1912
**Lowry, E. W.** .. .. *Can the Doctors work the Insurance Act ?* .. 1912
**Lucas, R. C.** .. .. *Points in Heredity* .. .. .. .. .. .. .. 1912
**McDonagh, J. E. R.** *Salvarsan in Syphilis and Allied Diseases* .. 1912
**McHattie, T. J. T.** .. *Educational Treatment of Stammering Children* 1911
**McKail, D.** .. .. *Public Health Chemistry and Bacteriology* .. 1912
**Major, S. D.** .. .. *Notabilia of Bath* .. .. .. .. .. .. .. (3) 1871
**Mark, L. P.** .. .. *Acromegaly : a Personal Experience* .. .. 1912
**Mayou, M. S.** .. *Diseases of the Eye* .. .. .. .. .. 2nd Ed. 1912
**Miles, A. Thomson and A.** *Manual of Surgery.* Vol. II. 4th Ed. 1912
**Miller, F. F.** .. .. *Physiology for the Young* .. .. .. .. .. (3) [1878]
**Minchin, W. C.** .. *Treatment of Tuberculosis, etc., with Allyl Sulphide* .. .. .. .. .. .. .. .. .. .. .. 1912
**Moll, A.** .. .. .. *The Sexual Life of the Child* (Trans. by E. Paul) 1912
**Morris, J. W.** (Ed.) *Handbook to Bath* .. .. .. .. .. .. (3) 1888
**Morris, Sir M.** .. *Diseases of the Skin* .. .. .. .. .. 5th Ed. 1911
**Morland, C. Riviere and E.** *Tuberculin Treatment* .. .. .. .. .. 1912
**Mulzer, P.** .. .. *The Therapy of Syphilis* (Trans. by A. Newbold) [N.D.]

**Murrell, W.** .. .. *What to do in Cases of Poisoning.* 11th Ed. 1912
**Newman, Sir A. Whitelegge and Sir G.** *Hygiene and Public Health* 12th Ed. 1911
**Paget, S.** .. .. *For and Against Experiments on Animals* .. 1912
*Pharmacopœia of the Queen's Hospital for Children* .. .. 5th Ed. 1911
**Pollock, and L. W. Harrison, C. E.** *Gonorrhœal Infections* .. .. 1912
*Provident Dispensary Work on National Insurance, The Light of Thirty Years of* .. .. .. .. .. .. 1911
**Rainey, R. Hutchison and H.** *Clinical Methods* .. .. 5th Ed. 1912
**Rawling, L. B.** .. *Landmarks and Surface Markings* .. 5th Ed. 1912
**Riviere and E. Morland, C.** *Tuberculin Treatment* .. .. .. .. 1912
**Roscoe, H. E.** .. *Spectrum Analysis* .. .. .. (3) 3rd Ed. 1873
**Rumford, B., Count of.** *Essays, Political, Economical and Philosophical.* 3 vols .. .. .. (3) New Ed. 1800–02
**Sewell, H.** .. .. *Quackery and Medical Law Reform* .. .. [1911]
**Still, G. F.** .. .. *Common Disorders and Diseases of Childhood.* 2nd Ed. 1912
**Thomson and A. Miles, A.** *Manual of Surgery.* Vol. II. 4th Ed. 1912
**Treves and A. Keith, Sir F.** *Surgical Applied Anatomy.* 6th Ed. 1911
**Tunstall, J.** .. .. *The Climate of Bath* .. .. .. .. .. .. (3) 1854
**Whiteford, C. H.** .. *An Operating Theatre in Private Practice* .. 1912
**Whitelegge and Sir G. Newman, Sir A.** *Hygiene and Public Health.* 12th Ed. 1911

### TRANSACTIONS, REPORTS, JOURNALS.

American Association of Genito-Urinary Surgeons, Transactions of the .. .. .. .. .. .. .. .. .. .. .. .. Vol. VI. 1911

American Journal of Obstetrics, The .. .. .. Vol. LXIV. 1911

American Journal of the Medical Sciences, The .. Vol. CXLII. 1911

American Laryngological Association, Transactions of the .. .. 1911

American Surgical Association, Transactions of the.. Vol. XXIX. 1911

Annales de Dermatologie et de Syphiligraphie .. Tom. XI. 1911

Archiv für klinische Chirurgie .. .. .. .. .. Bd. XCIV., XCV. 1911

Archives of Pediatrics .. .. .. .. .. .. .. .. Vol. XXVIII. 1911

Archives of the Middlesex Hospital .. .. .. (4) Vol. XXIII. 1911

Do. do. Index to Cancer Reports .. .. .. .. (4) 1911

Boston Medical and Surgical Journal, The .. Vol. CLXV. 1911

British Journal of Children's Diseases, The .. .. Vol. VIII. 1911

British Journal of Dermatology, The .. .. .. Vol. XXIII. 1911

Canadian Medical Association Journal, The .. .. .. Vol. I. 1911

Catalogue of Books, The English, for 1911 .. .. .. .. .. 1912

Deutsche Zeitschrift für Chirurgie .. .. .. Bds. CX., CXI. 1911

Edinburgh Medical Journal .. .. .. .. .. .. N.S., Vol. VII. 1911

Gazette des Hopitaux de Toulouse .. .. .. .. .. .. .. .. 1910

Glasgow Medical Journal, The .. .. .. .. .. Vol. LXXVI. 1911

Hospitals—

Guy's Hospital Reports .. .. .. .. .. .. Vol. LXV. 1911

St. Bartholomew's Hospital Reports .. .. Vol. XLVII. 1912

Indian Medical Gazette, The .. .. .. .. .. .. Vol. XLVI. 1911
Ireland, Transactions of the Royal Academy of Medicine in Vol. XXIX. 1911
Johns Hopkins Hospital Bulletin, The .. .. .. .. Vol. XXII. 1911
Journal of Experimental Medicine, The .. .. .. .. Vol. XIV. 1911
Journal of Laryngology, Rhinology and Otology, The Vol. XXVI. 1911
Journal of Mental Science, The .. .. .. .. .. .. Vol. LVII. 1911
Journal of Nervous and Mental Disease, The Vol. XXXVIII. 1911
Journal of the American Medical Association, The Vol. LVII. 1911
Journal of the Royal Sanitary Institute .. .. Vol. XXXII. 1911
Luzerne County Medical Society, Transactions of the Vol. XVIII. 1911
Medical Annual, The .. .. .. .. .. .. .. .. .. .. .. 1912
Medical Chronicle, The .. .. .. .. .. .. .. .. .. Vol. XXI. 1911
Medical Press and Circular, The .. .. .. Vol. CXLIII. 1911
Medical Review, The .. .. .. .. .. .. .. .. .. .. Vol. XIV. 1911
Ophthalmological Transactions .. .. .. .. .. Vol. XXXI. 1910–11
Philadelphia, Transactions of the College of Physicians of Vol. XXXIII. 1911
Practitioner, The .. .. .. .. .. .. .. .. .. .. Vol. LXXXVII. 1911
Public Health .. .. .. .. .. .. .. .. .. .. .. Vol. XXIV. 1910–11
Revue de Chirurgie.. .. .. .. .. .. .. .. .. .. Bd. XLIII. 1911
Revue générale d'Ophtalmologie .. .. .. .. .. Tom. XXX. 1911
Revue hebdomadaire de Laryngologie .. .. Tom. XXXI. 1911
Rhode Island Medical Society, Transactions of the Vol. VIII., Pt. I. 1910
Scalpel, The .. .. .. .. .. .. .. .. .. .. .. .. .. (3) Vol. I. 1823
Smithsonian Report, The, for 1910 .. .. .. .. .. .. .. .. 1911
Surgery, Gynecology and Obstetrics .. .. .. .. Vol. XIII. 1911
Therapeutic Gazette, The .. .. .. .. .. .. Vol. XXXV. 1911
Zentralblatt für Chirurgie .. .. .. .. .. .. .. .. .. .. .. 1911
Zentralblatt für Gynäkologie .. .. .. .. .. .. .. .. .. .. 1911
Zentralblatt für innere Medizin .. .. .. .. .. .. .. .. .. .. 1911
Zeitschrift für Urologie .. .. .. .. .. .. .. .. Bd. V. 1911

---

### MEETINGS OF SOCIETIES.

### Bristol Medico-Chirurgical Society.

*March 13th*, 1912.

Mr. C. A. Morton, President, in the Chair.

A discussion on "**The Intracranial Complications of Ear Disease**" (see p. 97).

---

*May 8th*, 1912.

Mr. C. A. Morton, President, in the Chair.

Dr. E. C. Williams: (1) A case of **Extensive hairy mole,**

### Local Medical Notes.

**Dr. Robert Fletcher.**—The January number of the *Index Medicus* records the fact that Dr. Robert Fletcher has, owing to the state of his health, withdrawn from the "engrossing editorial work" of this valuable publication after over thirty years' work in compiling the *Index Medicus.* Dr. Fletcher entered at the Bristol School in 1839, and it will be recalled that in 1910 the Council of the Royal College of Surgeons awarded him that rare distinction, their Honorary Medal. We wish Dr. Fletcher a speedy return to good health, and we note with satisfaction that the *Index Medicus* will continue the high standard of excellence it has attained in the hands of Dr. Fielding H. Garrison, who becomes the sole editor.

EXAMINATION SUCCESSES.

UNIVERSITY OF BRISTOL.—*M.B., Ch.B. Second Examination :* A. G. Bodman, D. G. C. Tasker, W. K. A. Richards, E. S. White, J. W. Gilbert.

UNIVERSITY OF LONDON.—*Second Examination for Medical Degrees. Part I :* A. W. Adams, F. K. Hayman. *Part II :* H. W. Cooke, G. M. Jackson, H. J. McCurrich, D. G. C. Tasker, W. E. Taylor, Mary E. Joll.

CONJOINT BOARD OF LONDON.—*Materia Medica :* H. B. Logan. *Medicine :* G. F. Fawn. *Surgery :* E. Christofferson,* G. Griffiths,* E. S. Abraham.*

L D.S.—*Mechanical Dentistry :* K. Batten, S. V. Shrimpton. *Final Examinations. Part I :* J. W. Gilbert. *Part II :* B. L. Morgan,* C. V. Osborne.*

**The Royal College of Physicians, London.**—Edward J. Cave, M.D. Lond., and J. Roger Charles, M.D. Cantab., have been elected Fellows of the College.

APPOINTMENTS.

Captain F. Percival Mackie, M.B., Ch.B. (Bristol), F.R.C.S., M.R.C.P., I.M.S., who was recently on the Royal Society's Sleeping Sickness Commission in Uganda, has been placed on special duty by the Government of India to investigate the disease known as Kala Azar. The appointment is under the recently-constituted Central Research Fund, and the inquiry will be carried on partly in the Madras Presidency and partly in Assam and Further India, where the disease is endemic.

C. A. Joll, M.S. Lond., F.R.C.S. Eng., has been appointed Senior Resident Medical Officer to Royal Free Hospital, Gray's Inn Road.

* Completes examination.

**Leprosy in South Africa.**—The now well-known dissemination of leprosy by means of the bed bug, as shown by the observations of Sandes in Robben Island Asylum, and by Long, working independently in Basutoland, who found that acid-fast bacilli can be found in nearly every bed bug which freely bit over leprous tissue, is illustrated by the experience of an old Bristol student, Dr. Alex. Spencer, of Middleburg. Giving an interesting story of his investigation of cases of leprosy in his district, he writes as follows :—

"The 'phone goes one morning early about two months ago. 'Are ye there ?' 'This is the Charge Office.' 'There 's a leper woman, brought in from the district last night, waiting to be seen here. Can you please make a point of seeing her as soon as possible this morning ?' The leper woman *was* a leper, and pretty bad. Her name was Kaitje Mamori, and she hied from a farm called Roodepoort No. 8 in this district. Her husband, she informed me, was also a leper at the Pretoria Leper Asylum, and his name Adonis ! So I certified her and put things in trim for the sending of her to Pretoria Asylum too in the leper coach, which must be wired for wherever it may be, perhaps picking up lepers in another part of the Transvaal. At home later I entered her up in my Register of Lepers—now twelve years old, and giving particulars of 112 sent to Pretoria from this district—and looked up 'Adonis.' He had been certified by me in August, 1902, and sent off to the Asylum. I also found three other lepers had been sent into town from this same farm, Roodepoort—two in 1907 and one in 1910—besides this Kaitje and Adonis. So I have to hie me to this farm, and get out a history and chart of all the belongings—wives, husbands and children—of all these lepers, connect them, try and trace the source of contagion, and examine all their belongings for any signs of the disease in any of them. When all this is done the report of the whole proceedings, step by step, is made out, and with a genealogical record of them forwarded to the Minister of the Interior and by him to the Asylum. On this occasion I found that the old man was a Mormon in practice, had four wives, each with six, six, seven and ten children respectively ! This is not out of the way for these native animals, and accounts for the rapid increase of 'em out of all proportion to Europeans, with which our laws do not allow us to compete. Of this family of twenty-nine, fourteen were (luckily) dead, mostly in infancy ; but the rest, many of them married and with hordes of children themselves, had all to be seen, scattered here and there all over the district, and examined for any signs of leprosy. So, too, with the other three cases removed more recently from this same farm suffering from the disease. This has occupied me at intervals during two months, and now is finished and the report gone in."

### *PUBLICATIONS RECEIVED.*

From Adlard and Son, London:
*The Bradshaw Lecture on some Points in Heredity.* By R. Clement Lucas, B.S., M.B. 1912. Price, 2s.

From George Allen & Company Ltd., London:
*The Sexual Life of the Child.* By Dr. Albert Moll. Translated from the German by Dr. Eden Paul. 1912. Price, 15s. net.

From Baillière, Tindall and Cox, London:
*The Cause of Cancer.* Being Part III of "Protozoa and Disease." By J. Jackson Clarke, M.B., F.R.C.S. 1912. Price, 7s. 6d. net.

From Cassell and Company Ltd., London:
*Clinical Methods.* By Robert Hutchison, M.D., and Harry Rainey, M.D. Fifth Edition. 1912. Price, 10s. 6d.

From J. & A. Churchill, London:
*The Educational Treatment of Stammering Children.* By Thomas J. T. McHattie, M.D. 1911. Price, 1s. net.
*Harelip and Cleft Palate.* By James Berry, B.S., F.R.C.S., and T. Percy Legg, M.S., F.R.C.S. 1912. Price, 12s. 6d. net.

From Henry Frowde and Hodder and Stoughton, London:
*Gonococcal Infections.* By Major C. E. Pollard and Major L. W. Harrison. 1912. Price, 5s. net.
*Tuberculin Treatment.* By Clive Riviere, M.D., and Egbert Morland, M.B., B.Sc. 1912. Price, 5s. net.
*Diseases of the Eye.* By M. Stephen Mayou, F.R.C.S. Second Edition. [1912.] Price, 5s. net.
*The Care of the Skin in Health.* By W. Allan Jamieson, M.D. 1912. Price, 2s. 6d. net.
*Common Disorders and Diseases of Childhood.* By George Frederic Still, M.A., M.D., F.R.C.P. Second Edition. 1912. Price, 16s. net.
*Salvarsan in Syphilis and Allied Diseases.* By J. E. R. McDonagh, F.R.C.S.,. 1912. Price, 7s. 6d. net.
*Manual of Surgery.* By Alexis Thomson, F.R.C.S. Edin., and Alexander Miles F.R.C.S. Edin. Fourth Edition. Vol. II. 1912. Price, 10s. 6d. net.

From Harrison and Sons, London:
*An Operating Theatre in Private Practice.* By C. Hamilton Whiteford, M.R.C.S. 1912.

From William Heinemann, London:
*Microbes and Toxins.* By Dr. Etienne Burnet. Translated from the French by Dr. Charles Broquet and W. M. Scott, M.D. 1912. Price, 5s. net.

From J. F. Lehmann, Munich:
*Die Chirurgischen Untersuchungsmethoden.* Von Prof. Dr. Hubert Gebele. 1912. Price, mk. 8.

From H. K. Lewis, London:
*The Pharmacopœia of the Queen's Hospital for Children.* Fifth Edition. 1911. Price, 2s. 6d. net.
*The New Sydenham Society.* Retrospective Memoranda. By Sir Jonathan Hutchinson, F.R.S. 1911. Price, 2s. 6d. net.
*What to do in Cases of Poisoning.* By William Murrell, M.D., F.R.C.P. Eleventh Edition. 1912. Price, 3s. net.
*For and Against Experiments on Animals.* By Stephen Paget, F.R.C.S. With an Introduction by the Right Hon. the Earl of Cromer, O.M. 1912. Price, 3s. 6d. net.
*Landmarks and Surface Markings of the Human Body.* By L. Bathe Rawling, M.B., B.C. Fifth Edition. 1912. Price, 5s. net.

From E. & S. Livingstone, Edinburgh:
*Fellowship Examination Papers, Edinburgh.* 1912. Price, 1s.
*Dental Examination Papers, Edinburgh.* [n.d.] Price, 1s.

From Longmans, Green and Co., London:
*A Manual of Surgical Treatment.* By Sir W. Watson Cheyne, Bart., C.B., F.R.C.S., and F. F. Burghard, M.S., F.R.C.S. New Edition, entirely revised and largely rewritten with the assistance of T. P. Legg, M.S., F.R.C.S., and Arthur Edmunds, M.S., F.R.C.S. Vol. II. 1912. Price, 21s. net.

From Rebman, Limited, London:
*The Therapy of Syphilis.* By Dr. Paul Mulzer. Translated by A. Newbold. [1912.] Price, 6s. net.

From The University Press, Manchester:
*Diseases of the Nervous System.* By Judson S. Bury, M.D. 1912. Price, 15s. net.

From Watts & Co., London:

*Can the Doctors work the Insurance Act?* By Ernest Ward Lowry, M.R.C.S., L.R.C.P. 1912. Price, 1s. net.

From John Wright and Sons Ltd., Bristol:
*An Index of Differential Diagnosis of Main Symptoms.* By Various Writers. Edited by Herbert French, M.A., M.D. 1912. Price, 30s. net.
*The Medical Annual.* 1912. Price, 8s. 6d. net.
*Consumption: Treatment at Home and Rules for Living.* By H. Warren Crowe, M.D. Third Edition. 1912. Price, 1s. net.
*Sprue: its Diagnosis and Treatment.* By Charles Begg, M.B., C.M. 1912. Price, 6s. net.
*The Etiology, Diagnosis and Prophylaxis of Pulmonary Phthisis.* By Alfred Harris, M.B., Ch.B., D.P.H. 1912. Price, 2s. 6d. net.
*Public Health Chemistry and Bacteriology.* By David McKail, M.D., D.P.H. 1912. Price, 6s. 6d. net.

